# The role of p16-cyclin D/CDK-pRb pathway in the tumorigenesis of endometrioid-type endometrial carcinoma

**DOI:** 10.1054/bjoc.1999.0980

**Published:** 2000-01-18

**Authors:** H Tsuda, K Yamamoto, T Inoue, I Uchiyama, N Umesaki

**Affiliations:** Departments of Obstetrics & Gynaecology and Pathology, Osaka City General Hospital, 2-13-22 Miyakojimahondori, Miyakojima, Osaka, 5340021, Japan; Department of Obstetrics & Gynaecology, Osaka City University Medical School, Miyakojima, Osaka, Japan

**Keywords:** p16^INK4^, cyclin D, CDK, pRb, endometrial cancer

## Abstract

We analysed p16 gene alteration and p16, cyclin-dependent kinase 4 (CDK4), CDK6, cyclin D1, cyclin D2, cyclin D3 and retinoblastoma protein (pRb) expression in ten normal endometriums (PE), 18 endometrial hyperplasias (EH) and 35 endometrial cancers (EC). Two of ten PE (20%), nine of 18 EH (50.0%) and 29 of 35 EC (82.9%) exhibited p16 nuclear staining. p16 expression was significantly higher in EC than EH (*P* = 0.0119). In the six p16 (–) EC, one was considered to have reduced gene dosage consistent with possible homozygous deletion of the *CDKN2* gene and three had methylation in 5′CpG island in the promoter region of the *p16* gene, whereas none showed such reduced gene dosage and four had methylation in the nine p16 (–) EH. Strong CDK4 staining was observed in 12 of 35 EC (34.3%) and one of 18 EH (5.6%). The strong expression of CDK4 was higher in EC than in EH (*P* = 0.0399). The expression of CDK4 was higher in EH than PE (*P* = 0.0054). The abnormalities of p16-cyclin D/CDK-pRb pathway were detected in 18 of 35 EC (51.4%). In conclusion, the expression of p16 and CDK4 may be an early event in the neoplastic transformation of endometrial cancer. © 2000 Cancer Research Campaign


British Journal of Cancer (2000) **82**(3), 675–682
© 2000 Cancer Research Campaign
DOI: 10.1054/ bjoc.1999.0980, available online at http://www.idealibrary.com on IDEAL®

### The role of p16-cyclin D/CDK-pRb pathway in the tumorigenesis of endometrioid-type endometrial carcinoma

**H Tsuda[1], K Yamamoto[1], T Inoue[2], I Uchiyama[2] and N Umesaki[3]**

Departments of [1]Obstetrics & Gynaecology and [2]Pathology, Osaka City General Hospital, 2-13-22 Miyakojimahondori, Miyakojima, Osaka 5340021, Japan; [3]Department of Obstetrics & Gynaecology, Osaka City University Medical School, Miyakojima, Osaka, Japan

**Summary** We analysed p16 gene alteration and p16, cyclin-dependent kinase 4 (CDK4), CDK6, cyclin D1, cyclin D2, cyclin D3 and retinoblastoma protein (pRb) expression in ten normal endometriums (PE), 18 endometrial hyperplasias (EH) and 35 endometrial cancers (EC). Two of ten PE (20%), nine of 18 EH (50.0%) and 29 of 35 EC (82.9%) exhibited p16 nuclear staining. p16 expression was significantly higher in EC than EH ($P$ = 0.0119). In the six p16 (–) EC, one was considered to have reduced gene dosage consistent with possible homozygous deletion of the *CDKN2* gene and three had methylation in 5′CpG island in the promoter region of the *p16* gene, whereas none showed such reduced gene dosage and four had methylation in the nine p16 (–) EH. Strong CDK4 staining was observed in 12 of 35 EC (34.3%) and one of 18 EH (5.6%). The strong expression of CDK4 was higher in EC than in EH ($P$ = 0.0399). The expression of CDK4 was higher in EH than PE ($P$ = 0.0054). The abnormalities of p16-cyclin D/CDK-pRb pathway were detected in 18 of 35 EC (51.4%). In conclusion, the expression of p16 and CDK4 may be an early event in the neoplastic transformation of endometrial cancer. © 2000 Cancer Research Campaign

**Keywords**: p16$^{INK4}$; cyclin D; CDK; pRb; endometrial cancer

Recent genetic and biochemical investigations of the molecular mechanisms governing the G1 to S progression in mammalian cells have demonstrated a central role for D-type cyclins and their partner kinases cyclin dependent kinase (CDK4) and CDK6 (Kamb et al, 1995; Sherr et al, 1995; Strauss et al, 1995; Weinberg et al, 1995; Hall et al, 1996). When activated by cyclin D, CDK is able to phosphorylate retinoblastoma protein (pRb) leading to the release of associated proteins like E2F that have the capability to activate genes necessary for cell progression through the G1 phase (Weinberg et al, 1995). p16 controls cell cycle proliferation during G1 by inhibiting the ability of cyclin D/CDK4 and cyclin D/CDK6 complexes to phosphorylate pRb (Serrano et al, 1993). The components of the p16-cyclin D/CDK-pRb pathway are frequently found to be altered in various types of cancer (Cirns et al, 1995; Nakagawa et al, 1995; Kinoshita et al, 1996; Barbieri et al, 1997; Zhang et al, 1997).

In solid tumours, there is a reciprocal correlation between genetic alterations of single members of the p16-cyclin D/CDK-pRb pathway (He et al, 1994; Schauer et al, 1994; Bartkova et al, 1996). It has been reported that hypophosphorylated active pRb can repress p16 expression, whereas inactivation of pRb by phosphorylation leads to p16 expression (Li et al, 1994). Consistent with these findings, *CDKN2* gene deletion and Rb deficiency are reported to be inversely correlated in many types of tumours (Bartkova et al, 1996; Kinoshita et al, 1996). In addition, a strong association between altered cyclin D1 and pRb expression has been reported in oesophageal tumours (Jiang et al, 1993). Muller et al have demonstrated that the cell cycle-dependent expression of cyclin D1 in tumour cell lines requires the presence of a functional pRb (Muller et al, 1994). The expression levels and activities of these proteins can modulate each other. It is thought to be important to evaluate these four elements simultaneously.

Endometrial carcinoma is the most common malignant neoplasm of the female genital tract; however, its molecular pathogenetic events are not fully understood. There are few reports about the abnormalities of p16-cyclin D/CDK-pRb pathway in endometrial cancer (Peiffer et al, 1995; Niemann et al, 1997; Shiozawa et al, 1997). Additionally, to our knowledge, there are no reports in which p16, cyclin D, CDK and pRb were examined simultaneously in endometrial cancer. In this study, we have examined all these key components of this G1 checkpoint mechanism by a combination of genetic and immunohistochemical approaches in normal endometrium (PE), endometrial hyperplasia (EH) and endometrial cancer (EC).

### MATERIALS AND METHODS

### Clinical samples

Paraffin-embedded tissue of 35 endometrioid-type endometrial cancers: (EC) (stage 1: 23; stage 2: five; stage 3: six; stage 4: none; recurrence: one) and 18 endometrial hyperplasia: (EH) (simple hyperplasia: 12, atypical hyperplasia: six) were collected at Osaka City General Hospital, Osaka, Japan. For control specimens, normal endometrial tissue (proliferative phase endometrium, PE) was selected from ten patients who had benign uterine leiomyoma. Histological diagnosis was confirmed by microscopic examination of the haematoxylin and eosin (H&E)-stained sections according

*Received 5 October 1998*
*Revised 8 September 1999*
*Accepted 9 September 1999*

*Correspondence to*: H Tsuda to World Health Organization criteria. Clinical stages were determined according to the International Federation of Gynecology and Obstetrics (FIGO) system. Regions of more than 80% tumour density were marked on H&E-stained slides to be used as guidelines for microdissection. Two 5-μm sections were cut from the paraffin block and the neoplastic tissue was microdissected away from contaminating normal tissue. The DNA was extracted using a DEXPAT™ kit (TaKaRa, Japan) and was finally precipitated in cold ethanol with sodium acetate and resuspended in Tris–EDTA buffer, pH 7.5.

### PCR-SSCP analysis

Polymerase chain reaction (PCR) was performed using 100 ng of genomic DNA as template in a 50 μl reaction volume containing 10 pmol of each rhodamine-labelled oligonucleotide primer, 5 μl of 10 × *Taq* DNA polymerase buffer (Perkin-Elmer, Cetus, CT, USA), 200 μM each dNTP (Perkin-Elmer, Cetus), 5% (v/v) dimethyl sulphoxide (DMSO) and 1.25 units *Taq* Gold DNA polymerase (Perkin-Elmer, Cetus). The primers used to amplify the regions of the *CDKN2* genes are the following (Hussussian et al, 1994): for exon 1, 5′-GGGAGCAGCATGGAGCCG-3′ (X1.31F)/5′-CTGGATCGG-CCTCCGACCGT-3′ (MK50) and 5′-AGCAGCATGGATCC GG-CGGCGG-3′ (MK49)/5′-AGTCGCCCGCCATCCCCT-3′ (X1.26R); for exon 2, 5′-AGCTTCCTTCCGTCATGC-3′ (X2.62F)/ 5′-GCAGCACCACAGCGTG3′ (286R), 5′-AGCCCAACT-GCGC-CGAC-3′ (200F)/5′-CCAGGTCCACGGGCAGA-3′ (346R) and 5′-TGGACGTGCGCGATGC-3′ (305F)/5′-GGAAGCTCTCAGG-GGTACAAATTC-3′ (X2.42R). After a 10 min initial denaturation step at 94°C, 40 cycles of 30 s at 94°C, 30 s at an annealing temperature (55–60°C), and 1 min at 72°C were performed in a thermal cycler (Perkin-Elmer, Cetus).

For single-strand conformational polymorphism (SSCP) analysis, 1 μl of each PCR product was mixed with 9 μl of SSCP loading buffer [98% (v/v) formamide, 10 mM EDTA] and incubated at 80°C for 5 min, followed by rapid cooling on ice. Two microlitres of the sample solution were loaded on a 6% acrylamide gel containing 1 × TBE (86 mM Tris–borate, 2 mM EDTA) and 5% glycerol. Following electrophoresis at 30 W at room temperature for 2–3 h, the gel was analysed using an FMBIO 100 fluorescent image analyser (TaKaRa, Japan). The presence of bands with variant migration pattern was confirmed by repeating PCR-SSCP at least once prior to extraction of the band for DNA sequence analysis.

### DNA sequencing

PCR products that revealed mobility shifts on SSCP analysis were cut from the gels and recovered. Re-amplification by PCR was followed by subcloning into pAMP1 vector (Gibco-BRL, MD, USA) and sequencing using M13/pUC primer. The sequence analysis for each product was performed with an ABI 310 genetic analyser (Perkin-Elmer, Cetus) and the Dye Terminator Cycle Sequencing FS Ready Reaction Kit (Perkin-Elmer, Cetus).

### Multiplex PCR of *CDKN2* gene

The *CDKN2* gene homozygous deletion was investigated by PCR for the ability to amplify a region of the gene compared with the ability to amplify, as an internal control, the human β-globin gene. The sequence of the primers used for multiplex PCR were *CDKN2* exon 2 (Marchini et al, 1997), sense 5′-TCTGACCATTCT-GTTCTCTC and antisense 5′-AGCACCACCAGCGTGTC; β-globin, sense 5′-CAACTTCATCCACGTTCACC and antisense 5′-GGTTGGCCAATCTACTCCCAGG. This p16 primer pair amplifies on the 5′ half of exon 2, where the majority of p16 mutations in human cancer cells have been described (Maestro et al, 1995). These primers amplify fragments of 166 bp and 205 bp respectively. One hundred nanograms of genomic DNA was amplified in a final volume of 50 μl containing 10 pmol of each oligonucleotide primer, 5 μl of 10 × *Taq* DNA polymerase buffer (Perkin-Elmer, Cetus), 200 μM each dNTP and 3.75 units *Taq* Gold DNA polymerase (Perkin-Elmer, Cetus). The cycles of amplification were as described previously (Kamb et al, 1994) and reduced to 30 cycles in the β-globin co-amplification to maintain the amplification in the exponential phase (Campbell et al, 1995). Samples were loaded on 4% MetaPhor agarose gel and visualized by ethidium bromide staining. The signal intensity was analysed using an FMBIO 100 fluorescent image analyser (TaKaRa, Japan). The *CDKN2* signal intensity was normalized against the β-globin signal intensity. We considered a tumour to have reduced gene dosage consistent with possible homozygous deletion of *CDKN2* gene in the tumour cells when the normalized *CDKN2* signal intensity in the tumour sample was reduced to less than 80% of that in the normal uterine control. In order to ascertain *CDKN2/p16* homozygous deletion, each experiment was repeated two times.

### Analysis of methylation in 5′CpG island in the promoter region of the *p16* gene

The methylation status of the 5′CpG island in the promoter region of the *p16* gene was determined with the CpGWiz™ p16 Methylation Kit (Oncor, Inc.). Briefly, 0.5–1.0 μg of DNA was denatured with 3 M sodium hydroxide at 50°C for 10 min and treated with sodium bisulphite following the manufacturer's protocol. After completion of the DNA modification, the DNA was purified by precipitation. The dissolved DNA was amplified by PCR, utilizing primers specific for the methylated (M) or unmethylated (U) sequences. A 5-μl aliquot of template (corresponding to treated DNA, positive control for methylated DNA, positive control for unmethylated sequences, and distilled water as negative control) was amplified in the presence of 10 × Universal PCR buffer, 2.5 mM dNTP mix, U or M primers, and AmpliTaq Gold™ (Perkin-Elmer), under the following conditions: preheating (95°C, 12 min), followed by 35 cycles (95°C, 45 s; 65°C, 45 s; 72°C, 1 min). The PCR product was analysed on a 2% agarose gel. The presence of DNA methylation was determined by the identification of one 145 bp fragment in those samples amplified with the M primers. All the cases were evaluated for the presence of an unmethylated specific fragment (154 bp), which served as an internal control for the quality of the treated DNA.

### Immunohistochemistry of p16, CDK4, CDK6, cyclin D1, cyclin D2, cyclin D3 and pRb proteins

Histological sections (4 μm) were affixed to glass slides, dewaxed and rehydrated. Autoclave unmasking process (10 min at 121°C in 10 mM citrate buffer, pH 6.0) was used. The sections were then incubated in 3% hydrogen peroxide for 10 min at room tempera-

 © 2000 Cancer Research Campaign

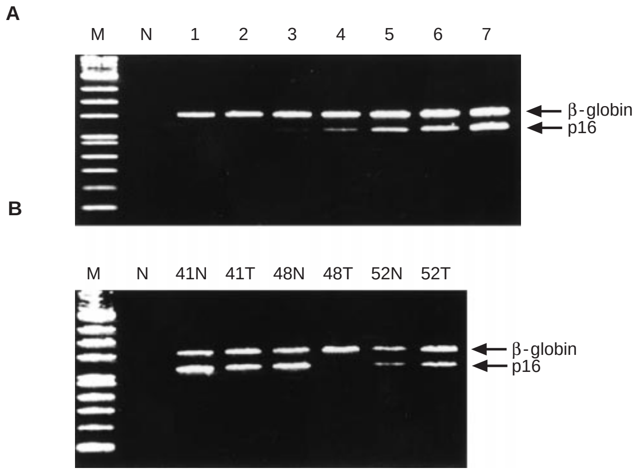

**Figure 1** (**A**) The comparative multiplex PCR assay using serial mixtures of normal human DNA and DNA from a central nervous system cell line (SF295) with homozygous deletion of *CDKN2/p16* locus. M: size marker; N: negative control; lane 1: SF295 DNA:normal DNA = 10:0; lane 2: 9.5:0.5; lane 3: 9:1; lane 4: 8:2; lane 5: 7:3; lane 6: 6:4; lane 7: 0:10. Reduction of *CDKN2/p16* amplification product was detected when normal DNA constituted less than 20% of the total DNA. (**B**) Multiplex PCR of *CDKN2/p16* locus in cases 41, 48 and 52. *CDKN2/p16* homozygous deletion was observed in case 48

ture to quench endogeneous peroxidase activity. The sections were reacted with one of the following primary antibodies at 4°C overnight: (a) rabbit anti-p16 polyclonal antibody (Pharmingen, San Diego, CA, USA), (b) mouse anti-pRb monoclonal antibody (Pharmingen), (c) rabbit anti-CDK4 polyclonal antibody (Santa Cruz Biotechnology, Inc., Santa Cruz, CA, USA), (d) rabbit anti-CDK6 polyclonal antibody (Santa Cruz Biotechnology, Inc.), (e) mouse anti-cyclin D1 monoclonal antibody (Medical and Biological Laboratories Co., Nagoya, Japan), (f) rabbit anti-cyclin D2 polyclonal antibody (Santa Cruz Biotechnology, Inc.), (g) rabbit anti-cyclin D3 polyclonal antibody (Santa Cruz Biotechnology, Inc.), or nonimmunized rabbit serum. After rinsing, the sections were incubated for 30 min with mouse or rabbit EnVision + Peroxidase (Dako, CA, USA). The peroxidase activity for p16, pRb, CDK4, CDK6, cyclin D1, cyclin D2 and cyclin D3 was visualized by applying diaminobenzidine chromogen containing 0.05% hydrogen peroxide for 2–10 min at room temperature. The sections were then counterstained with haematoxylin. Positive and negative control experiments were performed for each tumour staining.

### Interpretation of immunohistochemical staining

Evaluation of p16, pRb, CDK4, CDK6, cyclin D1, cyclin D2 and cyclin D3 positives was performed with semiquantitative analyses. The following scale was used: 0 = no immunoreactive tumour cells detectable or less than 5% of the tumour cells were positive with a weak intensity; 1 = 5–50% of the tumour cells were positive with a strong intensity; 2 = more than 50% of the tumour cells were positive with a strong intensity. Tumours were scored as p16-negative if less than 5% malignant cells had positive nuclear staining, and surrounding normal stromal cells showed adequate nuclear staining as a positive internal control. Tumours were regarded as p16-positive if more than 5% malignant cells had nuclear staining.

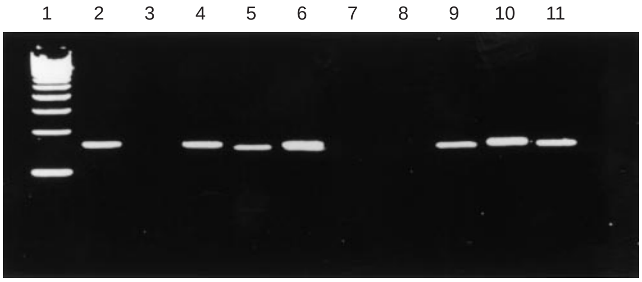

**Figure 2** Analysis of methylation in 5′CpG island in the promoter region of the *p16* gene. Primer sets used for amplification are designed as unmethylated (U) or methylated (M). Lane 1: size marker; lanes 2, 4, 6 and 8: PCR products with U primer in cases 2, 14, 24 and 37, respectively; lanes 3, 5, 7 and 9: PCR products with M primer in cases 2, 14, 24 and 37, respectively; lane 10: unmethylated DNA with U primer; lane 11: methylated DNA with M primer

Small lymphocytes, which showed no nuclear staining of p16, were used as a negative internal control (Kratzke et al, 1996). These criteria were used for evaluation of pRb staining. Negative nuclei staining (0) was considered as abnormal expression in p16 and pRb. Strong nuclear staining (2) was considered as abnormal expression of CDK4, CDK6, cyclin D1, cyclin D2 and cyclin D3 (Michalides et al, 1995).

### Statistical analysis

The abnormalities of p16, pRb, CDK4, CDK6, cyclin D1, cyclin D2 and cyclin D3 were analysed using Fisher's exact probability test to compare the different clinicopathologic groups with each other.

### RESULTS

### PCR-SSCP analysis of the *CDKN2* genes

SSCP analyses were performed for two *CDKN2* coding exons. One SSCP variant pattern was detected in assay for exon 1B in case 27. DNA sequence analysis of clones of this abnormally migrating SSCP fragment revealed a point mutation in intron 2. No mutations of either exons 1 or 2 were found in any samples (data not shown).

### Detection of homozygous deletion of the *CDKN2* genes

The comparative multiplex PCR assay was titrated using serial mixtures of normal human DNA and DNA from a CNS cell line (SF295) with homozygous deletion of *CDKN2*/p16 locus. Reduction of *CDKN2/p16* amplification product was indicated when normal DNA constituted less than 20% of the total DNA (Figure 1A). In case 48, *CDKN2/p16* homozygous deletion was observed (Figure 1B).

### Analysis of methylation in 5′CpG island in the promoter region of the *p16* gene

Forty-two samples DNA were available for p16 methylation analysis. Fifteen of 42 samples (35.7%) had methylation in 5′CpG island in the promoter region of the *p16* gene. Five of 15 EH (33.3%) and ten of 27 EC (37.0%) had methylation (Figure 2). Of

*© 2000 Cancer Research Campaign*

**Table 1** Endometrial hyperplasia

| Case | Age | Histology | SSCP | Methylation | HD | p16 | CDK4 | CDK6 | CCND1 | CCND2 | CCND3 | pRb |
|---|---|---|---|---|---|---|---|---|---|---|---|---|
| 1 | 78 | SH | N | M | N | 1 | 0 | 0 | 0 | 0 | 0 | 1 |
| 2 | 46 | SH | N | U | N | 1 | 1 | 0 | 0 | 0 | 0 | 1 |
| 3 | 66 | SH | N | U | N | 1 | 1 | 0 | 0 | 0 | 0 | 1 |
| 4 | 45 | SH | N | U | N | 1 | 1 | 0 | 0 | 0 | 0 | 1 |
| 5 | 49 | SH | N | ND | N | 1 | 1 | 1 | 0 | 0 | 0 | 1 |
| 6 | 46 | SH | N | U | N | 1 | 1 | 0 | 0 | 0 | 0 | 1 |
| 7 | 48 | SH | N | ND | N | 1 | 2 | 0 | 0 | 0 | 0 | 1 |
| 8 | 47 | SH | N | M | N | 0 | 0 | 0 | 0 | 0 | 0 | 1 |
| 9 | 52 | SH | N | M | N | 0 | 0 | 0 | 0 | 0 | 0 | 1 |
| 10 | 54 | SH | N | U | N | 0 | 1 | 0 | 0 | 0 | 0 | 1 |
| 11 | 70 | SH | N | ND | N | 0 | 1 | 0 | 0 | 0 | 0 | 2 |
| 12 | 51 | SH | N | U | N | 0 | 1 | 0 | 0 | 0 | 0 | 1 |
| 13 | 82 | AH | N | U | N | 1 | 1 | 0 | 0 | 0 | 0 | 1 |
| 14 | 83 | AH | N | M | N | 0 | 1 | 1 | 0 | 0 | 0 | 1 |
| 15 | 22 | AH | N | U | N | 1 | 1 | 0 | 0 | 0 | 0 | 1 |
| 16 | 52 | AH | N | U | N | 0 | 1 | 0 | 0 | 0 | 0 | 1 |
| 17 | 50 | AH | N | M | N | 0 | 1 | 0 | 0 | 0 | 0 | 2 |
| 18 | 74 | AH | N | U | N | 0 | 0 | 0 | 0 | 0 | 0 | 1 |

AH: atypical hyperplasia; SH: simple hyperplasia; N: normal; ND: not done.

**Table 2** Endometrial cancer

| Case | Age | Stage | Grade | SSCP | Methylation | HD | p16 | CDK4 | CDK6 | CCND1 | CCND2 | CCND3 | pRb |
|---|---|---|---|---|---|---|---|---|---|---|---|---|---|
| 19 | 59 | 1a | 1 | N | U | N | 1 | 0 | 0 | 0 | 0 | 0 | 2 |
| 20 | 39 | 1a | 1 | N | U | N | 1 | 2 | 0 | 0 | 0 | 0 | 1 |
| 21 | 53 | 1a | 2 | N | M | N | 1 | 1 | 0 | 0 | 0 | 0 | 1 |
| 22 | 55 | 1a | 2 | N | ND | N | 1 | 2 | 1 | 1 | 0 | 0 | 2 |
| 23 | 67 | 1a | 2 | N | M | N | 2 | 1 | 0 | 0 | 0 | 0 | 1 |
| 24 | 67 | 1b | 1 | N | U | N | 1 | 1 | 0 | 0 | 0 | 0 | 1 |
| 25 | 51 | 1b | 1 | N | M | N | 1 | 0 | 0 | 0 | 0 | 0 | 2 |
| 26 | 73 | 1b | 1 | N | U | N | 1 | 1 | 0 | 0 | 0 | 0 | 1 |
| 27 | 60 | 1b | 1 | A | U | N | 1 | 0 | 0 | 0 | 0 | 0 | 0 |
| 28 | 56 | 1b | 1 | N | U | N | 1 | 2 | 0 | 0 | 0 | 0 | 2 |
| 29 | 54 | 1b | 1 | N | U | N | 1 | 1 | 0 | 0 | 0 | 0 | 1 |
| 30 | 44 | 1b | 1 | N | ND | N | 1 | 2 | 0 | 0 | 0 | 0 | 2 |
| 31 | 59 | 1b | 2 | N | ND | N | 2 | 0 | 0 | 0 | 0 | 0 | 1 |
| 32 | 58 | 1b | 2 | N | M | N | 0 | 1 | 0 | 1 | 0 | 0 | 1 |
| 33 | 52 | 1b | 2 | N | U | N | 1 | 2 | 1 | 0 | 0 | 0 | 1 |
| 34 | 77 | 1b | 2 | N | U | N | 1 | 1 | 0 | 1 | 0 | 0 | 1 |
| 35 | 65 | 1b | 2 | N | U | N | 1 | 1 | 0 | 0 | 0 | 0 | 1 |
| 36 | 55 | 1b | 2 | N | U | N | 1 | 0 | 0 | 0 | 0 | 0 | 2 |
| 37 | 54 | 1b | 3 | N | M | N | 0 | 1 | 0 | 0 | 0 | 0 | 1 |
| 38 | 62 | 1b | 3 | N | ND | N | 2 | 2 | 0 | 0 | 0 | 0 | 0 |
| 39 | 42 | 1c | 1 | N | M | N | 1 | 1 | 0 | 1 | 0 | 0 | 1 |
| 40 | 54 | 1c | 2 | N | U | N | 1 | 0 | 0 | 1 | 0 | 0 | 1 |
| 41 | 52 | 1c | 2 | N | M | N | 0 | 0 | 0 | 1 | 0 | 0 | 0 |
| 42 | 45 | 2a | 1 | N | ND | N | 1 | 2 | 0 | 1 | 0 | 0 | 1 |
| 43 | 48 | 2a | 1 | N | M | N | 1 | 1 | 0 | 1 | 0 | 0 | 1 |
| 44 | 52 | 2a | 2 | N | ND | N | 1 | 2 | 0 | 1 | 0 | 0 | 1 |
| 45 | 68 | 2b | 1 | N | M | N | 1 | 1 | 0 | 0 | 0 | 0 | 2 |
| 46 | 80 | 2b | 3 | N | ND | N | 1 | 2 | 0 | 1 | 0 | 0 | 1 |
| 47 | 37 | 3a | 1 | N | U | N | 1 | 2 | 0 | 0 | 0 | 0 | 0 |
| 48 | 72 | 3a | 1 | – | ND | HD | 0 | 0 | 2 | 0 | 1 | 0 | 1 |
| 49 | 60 | 3a | 3 | N | U | N | 1 | 1 | 0 | 1 | 0 | 0 | 1 |
| 50 | 53 | 3c | 1 | N | U | N | 1 | 1 | 0 | 0 | 0 | 0 | 1 |
| 51 | 60 | 3c | 1 | N | U | N | 0 | 1 | 0 | 0 | 0 | 0 | 1 |
| 52 | 56 | 3c | 3 | N | U | N | 0 | 2 | 1 | 0 | 0 | 0 | 1 |
| 53 | 57 | RE | 2 | N | M | N | 1 | 2 | 0 | 0 | 0 | 0 | 1 |

N: normal; A: abnormal; U: unmethylated; M: methylated; ND: not done; HD: homozygous deletion

 *© 2000 Cancer Research Campaign*

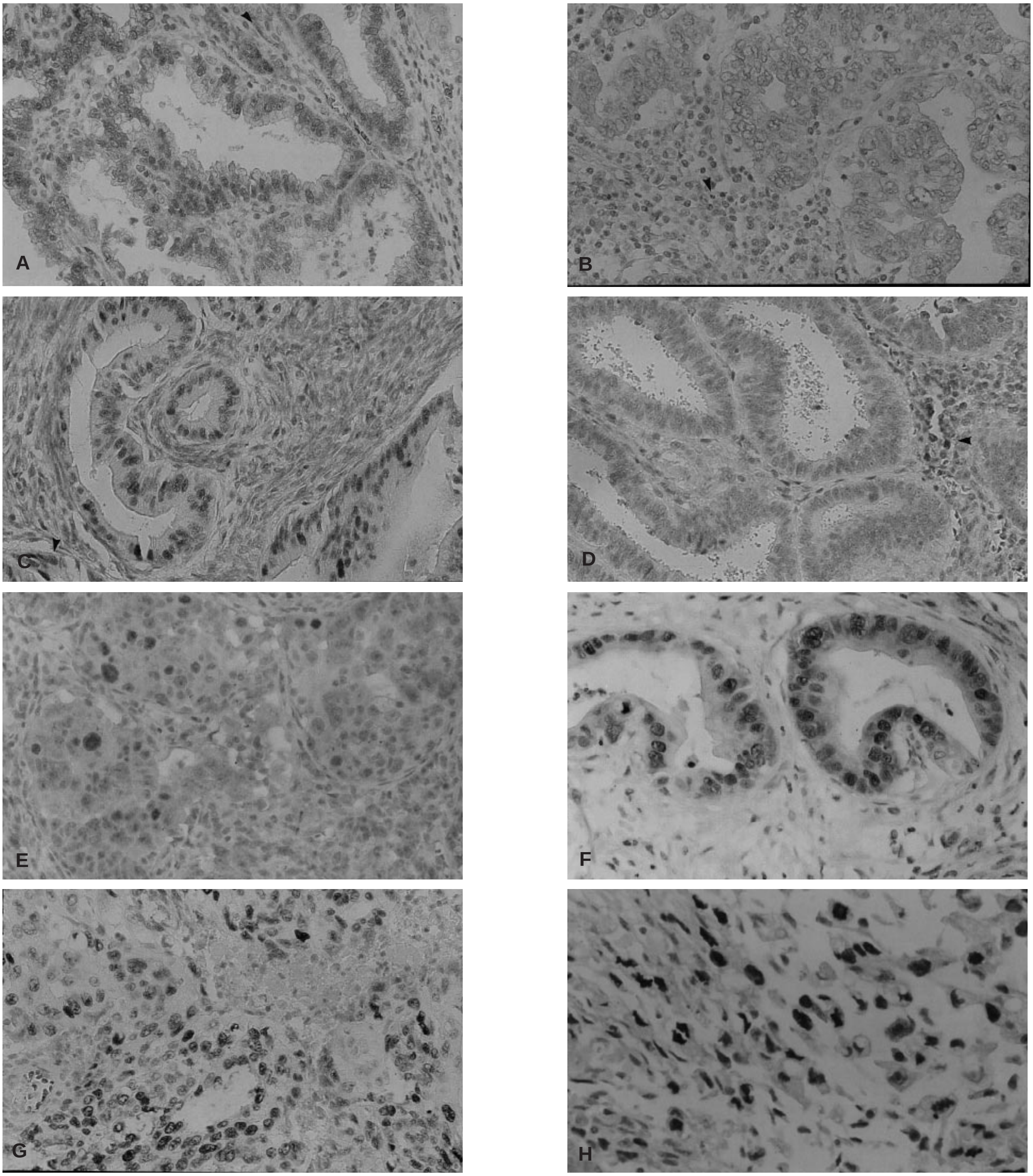

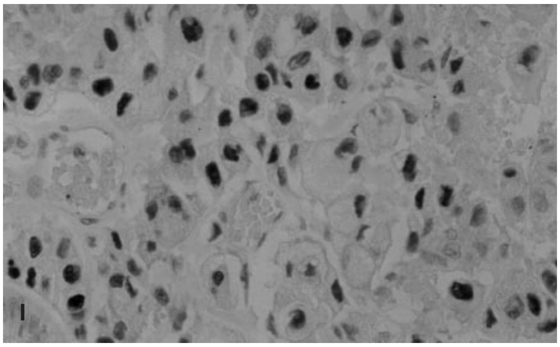

**Figure 3** Typical images of p16, pRb, CDK4, CDK6, cyclin D1, cyclin D2 and cyclin D3 staining. (**A**) Positive p16 immunostaining of case 47. (**B**) Negative p16 immunostaining of case 48. The section shows negative nuclear staining of the tumour cells. (**C**) Positive pRb immunostaining of case 48. (**D**) Negative pRb immunostaining of case 47. Note that stromal cells show distinct nuclear staining (arrow) of p16 and pRb, which provide positive internal controls for both proteins. (**E**) Strong positive CDK4 immunostaining in case 53. (**F**) Strong positive CDK6 immunostaining in case 48. (**G**) Strong positive cyclin D1 immunostaining in positive control. (**H**) Strong positive cyclin D2 immunostaining in positive control. (**I**) Strong positive cyclin D3 immunostaining in positive control. (**A–I**, original magnification 200 ×)

*© 2000 Cancer Research Campaign*

**Table 3** Correlation of expression of p16, CDK4, CDK6, CCND1, CCND2, CCND3 and pRb and clinicopathologic features

| Clinicopathologic features | NO | p16 | pRb | CDK4 1+ or 2+ | CDK4 2+ | CDK6 1+ or 2+ | CCND1 1+ | CCND2 1+ | CCND3 1+ | Pathway |
|---|---|---|---|---|---|---|---|---|---|---|
| Proliferative | 10 | 2 (20.0%) | 10 (100%) | 2 (20.0%)** | 0 (0%) | 0 (0%) | 0 (0%) | 0 (0%) | 0 (0%) | – |
| Hyperplasia | 18 | 9 (50.0%)* | 18 (100%) | 14 (77.8%)** | 1 (5.6%)*** | 2 (11.1%) | 0 (0%)**** | 0 (0%) | 0 (0%) | – |
| Cancer | 35 | 29 (82.9%)* | 31 (88.6%) | 27 (77.1%) | 12 (34.3%)*** | 4 (11.4%) | 10 (28.6%)**** | 1 (2.9%) | 0 (0%) | 18 (51.4%) |
| Clinical stage | | | | | | | | | | |
| I | 23 | 20 (87.0%) | 20 (87.0%) | 16 (69.6%) | 6 (26.1%) | 2 (8.7%) | 5 (21.7%) | 0 (0%) | 0 (0%) | 10 (43.5%) |
| II III RE | 12 | 9 (75.0%) | 11 (91.7%) | 11 (91.7%) | 6 (50.0%) | 2 (16.7%) | 5 (41.7%) | 1 (8.3%) | 0 (0%) | 8 (66.7%) |
| Histologic grade | | | | | | | | | | |
| G1 | 17 | 15 (88.2%) | 15 (88.2%) | 13 (76.4%) | 5 (29.4%) | 1 (5.9%) | 3 (17.6%) | 1 (5.9%) | 0 (0%) | 8 (47.1%) |
| G2 | 13 | 11 (84.6%) | 12 (92.3%) | 9 (69.2%) | 4 (30.8%) | 2 (15.4%) | 5 (38.5%) | 0 (0%) | 0 (0%) | 6 (46.2%) |
| G3 | 5 | 3 (60.0%) | 4 (80.0%) | 5 (100%) | 3 (60.0%) | 1 (20.0%) | 2 (40.0%) | 0 (0%) | 0 (0%) | 4 (80.0%) |

Pathway: p16-cyclin D/CDK-pRb pathway. *$P$ = 0.0119, **$P$ = 0.0054, ***$P$ = 0.039, ****$P$ = 0.0118.

nine p16 (–) EH and six p16 (–) EC, four (44.4%) and three (50.0%) had methylation respectively. Of five methylated EH, four (80%) cases had no expression of p16 and of ten methylated EC, three (30%) cases had no expression of p16.

### Immunohistochemistry of p16, pRb, CDK4, CDK6, cyclin D1, cyclin D2 and cyclin D3 proteins

The results of immunohistochemical analysis are shown in Tables 1 and 2. Two of ten PE (20%), nine of 18 EH (50.0%) and 29 of 35 EC (82.9%) had p16 nuclear staining. In the six p16 (–) EC, one was considered to have reduced gene dosage consistent with possible homozygous deletion of the *CDKN2* gene and three had methylation in 5′CpG island in the promoter region of the *p16* gene, wheres none showed such reduced gene dosage and four had methylation in the nine p16 (–) EH. p16 expression was significantly higher in EC than EH ($P$ = 0.0119). pRb was detectable in all ten of ten PE, all 18 of 18 EH and 31 of 35 EC; pRb was not detectable in four of 35 EC (11.4%). The EC cases with loss of either p16 or pRb expression are 1bG2, 1bG3, 1cG2, 3aG1, 3cG1, 3cG3, 1bG1, 1bG3, and 3aG1. Typical images of p16 and pRb staining are shown in Figure 3 A–D.

In total, two of ten PE (20%), 14 of 18 EH (77.8%) and 27 of 35 EC (77.1%) exhibited positive nuclear staining with CDK4 antibody. Strong CDK4 staining was observed in 12 of 35 EC (34.3%) and one of 18 EH (5.6%). The strong expression of CDK4 was higher in EC than in EH ($P$ = 0.0399). The weak and strong expression of CDK4 was higher in EH than PE ($P$ = 0.0054). The strong expression of CDK4 was higher in cases with grade 3 tumours (60.0%) than with grade 1 tumours (29.4%), but this difference was not significant. Ten cases of EC exhibited weak positive nuclear staining of cyclin D1 and its expression was higher in EC than EH ($P$ = 0.0118). In contrast, the expression of CDK6, cyclin D2 and cyclin D3 was rare in EC. Representative pictures of CDK4, CDK6, cyclin D1, cyclin D2 and cyclin D3 staining are shown in Figure 3 E–I. The abnormalities of p16-cyclin D/CDK-pRb pathway were detected in 18 of 35 EC (51.4%). Correlation of expression of p16, pRb, CDK4, CDK6, cyclin D1, cyclin D2 and cyclin D3 and clinicopathologic features are shown in Table 3.

### DISCUSSION

It was reported that the alterations of *CDKN2* were uncommon and late events in endometrial cancers (Peiffer et al, 1995; Milde-Langosch et al, 1999). In contrast, Shiozawa et al (1997) reported that in endometrial cancer, 65.8% of examined cases were negative for p16 protein. In our study, the rate of absence of p16 expression in EC was 17.1%. Based on our study, p16 alterations are thought to be rare in endometrial cancer. In our studies cases, the rate of absence of pRb expression in EC was low (11.4%). It was reported that alterations of pRb were rare events (Niemann et al, 1997; Milde-Langosch et al, 1999). In particular, Yaginuma et al (1996) demonstrated that Rb gene abnormality was a rare event in endometrial cancer. Geradts et al (1996) reported that studies on the expression of Rb in neoplasms will include an unknown, but probably small, number of positive stains despite the lack of functional pRb. The pRb alterations are thought to be rare in endometrial cancer. Further examination will be required to solve this problem.

There were no cases exhibiting overexpression of cyclin D1 in this study. Cyclin D1 was reported to be overexpressed in 30–50% of invasive primary breast cancers and to play a key role in mediating mitogenic response to steroids and growth factor in breast cancer cells in vitro (Buckley et al, 1993; Dutta et al, 1995; McIntosh et al, 1995). In addition, it was reported that the abnormal expression of tumour suppressor genes such as p16 or pRb was present in 40% of breast cancer (Cairns et al, 1995; Herman et al, 1995; Pietilainen et al, 1995). Both breast cancer and endometrial cancer are sex steroid-dependent tumours. Studies in mice have demonstrated that cyclin D1 plays a pivotal role in normal mammary gland development. Cyclin D1 knockout mice fail to undergo the lobular proliferative changes normally seen in the breast epithelial compartment under the influence of steroid hormones in pregnancy (Sicinski et al, 1995), whereas overexpression of cyclin D1 in the mammary gland of transgenic animals results in premature lobulo-alveolar development, abnormal epithelial proliferation in pregnancy, and the late development of adenocarcinoma (Wang et al, 1994). It was demonstrated that cyclin D1 and oestrogen receptor (ER) gene expression are positively correlated in primary breast cancer (Zuberberg et al, 1995; Hui et al, 1996) and that oestrogen stimulation increases cyclin D1 gene expression in an ER-positive cultured breast cancer cell line (Prall et al, 1997). The mechanism of development and carcinogenesis of endometrium may differ from that of mammary gland tissue.

In this study, expression of CDK4 significantly increased from PE to EC. However, it did not significantly increase with the

© 2000 Cancer Research Campaign progression from stage 1 to 3. These data suggest that the overexpression of CDK4 protein is important in the early stages of endometrial oncogenesis.

The meaning of loss of p16 expression in EH remains unclear. The loss of p16 protein in EH may be precursors to tumorigenesis. Four of nine p16 (–) EH (44.4%) had methylation in 5′CpG island in the promoter region of the *p16* gene. The methylation of the 5′CpG island of *p16/CDKN2* is thought to be associated with inactivation of this tumour suppressor gene. However, the fact that the loss of p16 protein was significantly higher in EH than EC is contradictory. It is possible that in EH, the loss of p16 protein with methylation may differ from that without methylation. Another speculation is that the loss of p16 protein may be involved in normal cell function. The loss of p16 protein was significantly higher in EH than in EC and one of the six p16 (–) EC was considered to have reduced gene dosage consistent with possible homozygous deletion of the *CDKN2* gene, whereas none of the nine p16 (–) EH showed such reduced gene dosage. The fact that five of 15 EH (33.3%) had methylation in 5′CpG island in the promoter region of the *p16* gene may be contradictory; however, Gonzalez reported that *p16/CDKN2* was methylated and not expressed in the 50% of normal colon tissue (Gonzalez et al, 1995). It has been reported that overexpression of p16 might be an important early event in neoplastic transformation (Bartkova et al, 1996; Shiozawa et al, 1997; Shigemasa et al, 1997). Yao et al (1998) reported that primary soft tissue sarcoma overexpressing CDK4 also had high levels of pRb and/or p16, which may reflect the onset of feedback to counteract the overexpression of CDK4. It may be that the loss of p16 in EH is a reversible change of the feedback system between CDK4 and p16. Further examination will be required to determine if the transcriptional silencing of *p16/CDKN2* observed in EH is involved in normal cell function or is a precursor to tumorigenesis.

In conclusion, the expression of p16 and CDK4 may be an early event in the neoplastic transformation of endometrial cancer.

### ACKNOWLEDGEMENTS

We are grateful to Dr Kazuhiko Nakagawa of the Kinki University and Ms Kathrine Allikian of Enzyme Bio-Systems, Ltd for their critical review of this article, and Mr Yuzuru Yasuji, Ms Fumi Kaibara and Ms Keiko Higa for their technical assistance. This work was supported by grants from the Osaka City General Hospital.

### REFERENCES

Barbieri F, Cagnoli M, Ragni N, Pedulla F, Foglia G and Alama A (1997) Expression of cyclin D1 correlates with malignancy in human ovarian tumours. *Br J Cancer* **75**: 1263–1268

Bartkova J, Lukas J, Guldberg P, Alsner J, Kirkin AF, Zeuthen J and Bartek J (1996) The p16-cyclin D/Cdk4-pRb pathway as a functional unit frequently altered in melanoma pathogenesis. *Cancer Res* **56**: 5475–5483

Buckley M, Sweeney KJE, Hamilton JA, Sini RL, Manning DL, Nicholson RI, deFazio A, Watts CKW, Musgrove EA and Sutherland RL (1993) Expression and amplification of cyclin genes in human breast cancer. *Oncogene* **8**: 2127–2133

Cairns P, Poladcik TJ, Eby Y, Tokino K, Califano J, Merlo A, Mao L, Herath J, Jenkins R and Westra W (1995) Frequency of homozygous deletion at p16/CDKN2 in primary human tumors. *Nat Genet* **11**: 210–212

Campbell IG, Beynon G, Davis M and Englefield P (1995) LOH and mutation analysis of CDKN2 in primary human ovarian cancer. *Int J Cancer* **63**: 222–225

Cirns P et al (1995) Frequency of homozygous deletion at p16/CDKN2 in primary human tumors. *Nat Genet* **11**: 210–212

Dutta A, Chandra R, Leiter LM and Lester S (1995) Cyclins as markers of tumor proliferation: immunocytochemical studies in breast cancer. *Proc Natl Acad Sci USA* **92**: 5386–5390

Geradts J, Kratzke RA, Crush-Stanton S, Wen SF and Lincoln CE (1996) Wild-type and mutant retinoblastoma protein in paraffin sections. *Mod Pathol* **9**: 339–347

Gillett C et al (1996) Cyclin D1 and prognosis in breast cancer. *Int J Cancer* **69**: 92–99

Gnozalez-Zulueta M, Bender CM, Yang AS, Nguyen T, Beart RW, Van Tornout JM and Jones PA (1995) Methylation of the 5′CpG island of the p16/CDKN2 tumor suppressor gene in normal and transformed human tissues correlates with gene silencing. *Cancer Res* **55**: 4531–4535

Hall M and Peters G (1996) Genetic alterations of cyclins, cyclin-dependent kinases, and Cdk inhibitors in human cancer. *Adv Cancer Res* **68**: 67–108

He J, Allen JR, Collins VP, Allalunis-Turner MJ, Godbout R, Day RS and James CD (1994) CDK4 amplification is an alternative mechanism to p16 homozygous deletion in glioma cell lines. *Cancer Res* **54**: 5804–5807

Herman JG, Merlo A, Mao L, Lapidus RG, Issa J-PJ, Davidson NE, Sidransky D and Baylin SB (1995) Inactivation of the CDKN2/p16/MTS1 gene is frequently associated with aberrant DNA methylation in all common human cancers. *Cancer Res* **55**: 4525–4530

Hui R, Cornish AL, McClelland RA, Robertson JFR, Blamey RW, Musgrove EA, Nicholson RI and Sutherland RL (1996) Cyclin D1 and estrogen receptor messenger RNA levels are positively correlated in primary breast cancer. *Clin Cancer Res* **2**: 923–928

Hussussian CJ, Struewing JP, Goldstein AM, Higgins PAT and Ally DS (1994) Germline p16 mutations in familial melanoma. *Nature Genet* **8**: 15–21

Jiang W, Zhang YJ, Kahn SM, Hollstein MC, Santella RM, Lu SH, Harris CC, Montesano R and Weinstein IB (1993) Altered expression of the cyclin D1 and retinoblastoma genes in human esophageal cancer. *Proc Natl Acad Sci USA* **90**: 9026–9030

Kamb A, Gruis NA, Weaver-Feldhaus J, Liu Q, Harshman K, Tavtigian SV, Stocker E, Day RS, III, Johnson BE and Skolnick MH (1994) A cell cycle regulator potentially involved in genesis of many tumor types. *Science* **264**: 436–440

Kamb A (1995) Cell-cycle regulators and cancer. *Trends Genet* **11**: 136–140

Kinoshita I, Dosaka-Akita H, Mishina T, Akie K, Nishi M, Hiroumi H, Hommura F and Kawakami Y (1996) Altered $p16^{INK4}$ and retinoblastoma protein status in non-small cell lung cancer: potential synergistic effect with altered p53 protein on proliferative activity. *Cancer Res* **56**: 5557–5562

Kratzke RA, Greatens TM, Rubins JB, Maddaus MA, Niewoehner DE, Niehans GA and Geradts J (1996) Rb and $p16^{INK4A}$ expression in resected non-small cell lung tumors. *Cancer Res* **56**: 3415–3420

Li Y, Nicholos MA, Shay JW and Xiong Y (1994) Transcriptional repression of the D-type cyclin-dependent kinase inhibitor p16 by the retinoblastoma susceptibility gene product pRB. *Cancer Res* **54**: 6078–6082

McIntosh GG, Anderson JJ, Milton I, Steward M, Parr AH, Thomas MD, Henry JA, Angus B, Lennard TWJ and Horne CHW (1995) Determination of the prognostic value of cyclin D1 overexpression in breast cancer. *Oncogene* **11**: 885–891

Maestro R and Boiocchi M (1995) Sunlight and melanoma: an answer from MTS1 (p16). *Science* **267**: 15–16

Marchini S, Codegoni AM, Bonazzi C, Chiari S and Broggini M (1997) Absence of deletions but frequent loss of expression of $p16^{INK4}$ in human ovarian tumours. *Br J Cancer* **76**: 146–149

Michalides R, van Veelen N, Hart A, Loftus B, Wientjens E and Balm A (1995) Overexpression of cyclin D1 correlates with recurrence in a group of forty-seven operable squmaous cell carcinomas of the head and neck. *Cancer Res* **55**: 975–978

Milde-Langosch K, Riethdorf L, Bamberger AM and Loning T (1999) P16/MTS1 and pRB expression in endometrial carcinomas. *Virchows Arch* **434**: 23–28

Muller H, Lukas J, Schneider A, Warthoe P, Bartek J, Eilers M and Strauss M (1994) Cyclin D1 expression is regulated by the retinoblastoma protein. *Proc Natl Acad Sci USA* **91**: 2945–2949

Nakagawa K, Conrad NK, Williams JP, Johnson BE and Kelly MJ (1995) Mechanism of inactivation of CDKN2 and MTS2 in non-small-cell lung cancer and association with advanced stage. *Oncogene* **11**: 1843–1851

Niemann TH, Yilmaz AG, McGaughy VR and Vaccarello L (1997) Retinoblastoma protein expression in endometrial carcinoma. *Gynecol Oncol* **65**: 232–236

Peiffer SL, Bartsch D, Whelan AJ, Mutch DG, Herzog TJ and Goodfellow PJ (1995) Low frequency of CDKN2 mutation in endometrial carcinomas. *Mol Carcinog* **13**: 210–212

*© 2000 Cancer Research Campaign*

Pietilainen T, Lipponen P, Aaltomaa S, Eskelinen M, Kosma VM and Syrjanen K (1995) Expression of retinoblastoma gene protein (Rb) in breast cancer as related to established prognostic factors and survival. *Eur J Cancer* **31A**: 329–333

Prall OWJ, Sarcevic B, Musgrove EA, Watts CKW and Sutherland RL (1997) Estrogen-induced activation of Cdk4 and Cdk3 during G1-S phase progression is accompanied by increased cyclin D1 expression and decreased cyclin-dependent kinase inhibitor association with cyclin E-Cdk2. *J Biol Chem* **272**: 10882–10894

Schauer IE, Siriwardana S, Langan TA and Sclafani RI (1994) Cyclin D1 overexpression vs. retinoblastoma inactivation: implications for growth control evasion in non-small and small cell lung cancer. *Proc Natl Acad Sci USA* **91**: 7827–7831

Serrano M, Hannon GJ and Beach D (1993) A new regulatory motif in cell-cycle control causing specific inhibition of cyclinD/CDK4. *Nature* **366**: 704–707

Sherr CJ and Roberts JM (1995) Inhibitors of mammalian G1 cyclin-dependent kinases. *Gene Dev* **9**: 1149–1163

Shigemasa K, Hu C, West CM, Clarke J, Parham GP, Parmley TH, Korourian S, Baker VV and O'Brien TJ. (1997) p16 overexpression: a potential early indicator of transformation in ovarian carcinoma. *J Soc Gynecol Invest* **4**: 95–102

Shiozawa T, Nikaido T, Shimizu M, Zhai Y and Fujii S (1997) Immunohistochemical analysis of the expression of CDK4 and $p16^{INK4}$ in human endometrioid-type endometrial carcinoma. *Cancer* **80**: 2250–2256

Sicinski P, Donaher JL, Parker SB, Li T, Fazeli A, Gardner H, Haslam SZ, Bronson RT, Elledge SJ and Weinberg RA (1995) Cyclin D1 provides a link between development and oncogenesis in the retina and breast. *Cell* **82**: 621–630

Strauss M, Lukas J and Bartek J (1995) Unrestricted cell cycling and cancer. *Nat Med* **1**: 1245–1246

Wang TC, Cardiff RD, Zukerberg L, Lees E, Arnold A and Schmidt EV (1994) Mammary hyperplasia and carcinoma in MMTV-cyclin D1 transgenic mice. *Nature (Lond)* **369**: 669–671

Weinberg RA (1995) The retinoblastoma protein and cell cycle control. *Cell* **81**: 323–330

Yao J, Pollock RE, Lang A, Ming T, Pisters PWT, Goodrich D, El-Naggar A and Yu Dihua (1998) Infrequent mutation of the p16/MTS1 gene and overexpression of cyclin-dependent kinase 4 in human primary soft-tissue sarcoma. *Clin Cancer Res* **4**: 1065–1070

Yaginuma Y, Katayama H, Kawai K, Duenas JC and Ishikawa M (1996) Analysis of the retinoblastoma gene in human endometrial carcinoma. *Obstet Gynecol* **87**: 755–759

Zhang T, Nanney LB, Luongo C, Lamps, Heppner KJ, DuBois RN and Beauchamp RD (1997) Concurrent overexpression of cyclinD1 and cyclin-dependent kinase 4 (Cdk4) in intestinal adenomas from multiple intestinal neoplasia (Min) mice and human familial adenomatous polyposis patients. *Cancer Res* **57**: 169–175

Zuberberg LR, Yang WI, Gadd M, Thor AD, Koerner FC, Schmidt EV and Arnold A (1995) Cyclin D1 (PRAD1) protein expression in breast cancer: approximately one-third of infiltrating mammary carcinomas show overexpression of the cyclin D1 oncogene. *Mod Pathol* **8**: 560–566

 © 2000 Cancer Research Campaign
